# The data on psychological adaptation during polar winter-overs in Sub-Antarctic and Antarctic stations

**DOI:** 10.1016/j.dib.2020.105324

**Published:** 2020-02-25

**Authors:** Michel Nicolas, Guillaume Martinent, Peter Suedfeld, Marvin Gaudino

**Affiliations:** a University of Bourgogne Franche-Comté, Laboratory Psy-DREPI (EA 7458), Dijon, France; b University of Lyon, University of Claude Bernard Lyon 1, Laboratory of Vulnerability and Innovation in Sport, France; c Department of Psychology, The University of British Columbia, Vancouver, Canada

**Keywords:** Emotional changes, Extreme environment, Isolated and confined environment, Occupational investment, Physical fatigue, Polar stations, Psychological adaptation, Social relationships

## Abstract

The data presented in this article relate to the research article entitled “assessing psychological adaptation during polar winter-overs: The isolated and confined environments questionnaire (ICE-Q)” [1]. These data were acquired in order to develop a standardized instrument – the ICE-Q – designed to assess psychological adaptation within isolated, confined, and extreme environments. A total of 140 winterers from several sub-Antarctic (Amsterdam, Crozet, Kerguelen) and Antarctic (Concordia, Terre Adélie) stations voluntarily participated. Data were collected by multiple self-report questionnaires including a wide variety of well-known and validated questionnaires to record the winterers’ responses to polar stations. Data were gathered across two or three winter seasons within each of the 5 polar stations to ensure sufficiently large sample. From four to seven measurement time along a one-year period were proposed to the participants, resulting in 479 momentary assessments. Results of exploratory factor analyses, confirmatory factor analyses, exploratory structural equation modelling, reliability analyses, and test-retest provided strong evidence for the construct validity of the ICE-Q (19–item 4-factor questionnaire). The four factors were social, emotional, occupational and physical. Future studies would examine the dynamic of psychological adaptation in isolated, confined and/or extreme environments during polar missions.

**Table:** Specifications Table

Subject	Applied psychology
Specific subject area	Environmental psychology, health psychology, psychological adaptation.
Type of data	Tables and Figures
How data were acquired	Self-reported data was collected from winterers during winter season mission in sub-Antarctic or Antarctic stations
Data format	Analyzed
Parameters for data collection	Data on psychological adaptation during sub-Antarctic or Antarctic missions were obtained using self-report questionnaires
Description of data collection	Data were collected by multiple self-report questionnaires assessing winterers' responses to polar stations across several measurement points during sub-Antarctic and Antarctic missions
Data source location	Dijon, France.
Data accessibility	Data are included in this article
Related research article	Nicolas, M., Martinent, G., Gaudino, M., & Suedfeld, P, Assessing psychological adaptation during polar winter-overs: The isolated and confined environments questionnaire (ICE-Q), J Environ Psychol. https://doi.org/10.1016/j.jenvp.2019.101317 [1]

**Table:** 

**Value of the Data** • The data provides a short, quick, cost-effective and non-invasive measure to monitor psychological adaptation within the four key domains (social, emotional, occupational and physical) in isolated, confined and extreme environment. • The data can help psychologists' to monitor psychological adaptation in extreme environment and to assist individuals optimizing their well-being and performance in such isolated, confined and extreme environment. • The data can help researchers for understanding of factors influencing the adaptation to isolated, confined and extreme environment. • The findings of the present data call for further research to examine the dynamic of psychological adaptation in extreme environment.

## 1 Data

The data presented in this article is complementary to the research article entitled “assessing psychological adaptation during polar winter-overs: The isolated and confined environments questionnaire (ICE-Q)” [1]. A total of 140 winterers (*M*age = 34.42 ± 13.34 years, 17.9% of females) voluntarily participated. Participants were members of 14 distinct polar missions from several sub-Antarctic (Amsterdam, Crozet, Kerguelen) and Antarctic (Concordia, Terre Adélie) stations. Data were gathered during several winter seasons and in several polar stations to ensure sufficiently large sample (no winterer participated in more than one winter season). Table 1 detailed the year of data gathering, the place of the polar mission and the number of participants. Table 2 indicates the initial questionnaire with the content of the items except the questionnaire protected by copyright.

**Table 1:** Descriptive statistics of the several polar missions.

Years of data gathering	Place of the polar mission	Number of participants	Gender (*N* females and *N* males)
2010–2011	Concordia	13	1 female and 12 males
2011–2012	Amsterdam	8	3 females and 5 males
2011–2012	Crozet	12	2 females and 10 males
2011–2012	Kerguelen	11	11 males
2012–2013	Amsterdam	4	1 female and 3 males
2012–2013	Concordia	15	4 females and 11 males
2012–2013	Crozet	4	2 females and 2 males
2012–2013	Kerguelen	1	1 female
2012–2013	Terre Adélie	11	2 females and 9 males
2013–2014	Amsterdam	10	1 female and 9 males
2013–2014	Concordia	11	11 males
2013–2014	Crozet	10	4 females and 6 males
2013–2014	Kerguelen	16	2 females and 14 males
2013–2014	Terre Adélie	14	2 females and 12 males

**Table 2:** Description of the initial questionnaire and content of the items.

Item	Dimension	Questionnaire	Formulation
1	Fatigue	RESTQ	Copyright protected
2	Fatigue	RESTQ	Copyright protected
3	Fatigue	RESTQ	Copyright protected
4	Lack of energy	RESTQ	Copyright protected
5	Lack of energy	RESTQ	Copyright protected
6	Lack of energy	RESTQ	Copyright protected
7	General stress	RESTQ	Copyright protected
8	General stress	RESTQ	Copyright protected
9	General stress	RESTQ	Copyright protected
10	Social stress	RESTQ	Copyright protected
11	Social stress	RESTQ	Copyright protected
12	Social stress	RESTQ	Copyright protected
13	Emotional stress	RESTQ	Copyright protected
14	Emotional stress	RESTQ	Copyright protected
15	Emotional stress	RESTQ	Copyright protected
16	Conflicts/Pressure	RESTQ	Copyright protected
17	Conflicts/Pressure	RESTQ	Copyright protected
18	Conflicts/Pressure	RESTQ	Copyright protected
19	Physical Complaints	RESTQ	Copyright protected
20	Physical Complaints	RESTQ	Copyright protected
21	Physical Complaints	RESTQ	Copyright protected
22	Success	RESTQ	Copyright protected
23	Success	RESTQ	Copyright protected
24	Success	RESTQ	Copyright protected
25	Sleep Quality	RESTQ	Copyright protected
26	Sleep Quality	RESTQ	Copyright protected
27	Sleep Quality	RESTQ	Copyright protected
28	Physical Recovery	RESTQ	Copyright protected
29	Physical Recovery	RESTQ	Copyright protected
30	Physical Recovery	RESTQ	Copyright protected
31	General Well–being	RESTQ	Copyright protected
32	General Well–being	RESTQ	Copyright protected
33	General Well–being	RESTQ	Copyright protected
34	Social Recovery	RESTQ	Copyright protected
35	Social Recovery	RESTQ	Copyright protected
36	Social Recovery	RESTQ	Copyright protected
37	Cohesiveness	GES	Group members feel a sense of belongingness to the group
38	Cohesiveness	GES	Group members show that they care for one another
39	Cohesiveness	GES	Group members can understand what others in the group are going through
40	Cohesiveness	GES	Group members are supportive of one another
41	Cohesiveness	GES	Group members encourage each other in reaching their goals
42	Implementation–Preparedness	GES	The rules of the group are clearly understood by the members
43	Implementation–Preparedness	GES	The activities of the group are planned
44	Implementation–Preparedness	GES	Group activities are easy to follow
45	Implementation–Preparedness	GES	Group members learn new ways of solving problems
46	Implementation–Preparedness	GES	Group members are encouraged to act autonomously
47	Counterproductive Activity	GES	Group members sometimes yell at each other
48	Counterproductive Activity	GES	Group members are engaged in petty quarrels with one another
49	Counterproductive Activity	GES	The atmosphere of the group is often hostile
50	Counterproductive Activity	GES	There seems to be a lot of tension between group members
51	Decision Latitude	JCQ	My job requires me to be creative
52	Decision Latitude	JCQ	My job allows me to make a lot of decisions on my own
53	Decision Latitude	JCQ	I have a lot of say about what happens on my job
54	Decision Latitude	JCQ	I get to do a variety of different things on my job
55	Decision Latitude	JCQ	I have an opportunity to develop my own special abilities
56	job demands	JCQ	My job requires working very fast
57	job demands	JCQ	My job requires working very hard
58	job demands	JCQ	I receive conflicting demands that others make
59	job demands	JCQ	I am asked to do an excessive amount of work
60	job demands	JCQ	My job requires long periods of intense concentration on the task
61	Coworker support	JCQ	People I work with are competent in doing their jobs
62	Coworker support	JCQ	People I work with take a personal interest in me
63	Coworker support	JCQ	People I work with are friendly
64	Coworker support	JCQ	People I work with are helpful in getting the job done
65	Supervisor support	JCQ	My supervisor (or colleague) is concerned about the welfare of those under him
66	Supervisor support	JCQ	My supervisor (or colleague) pays attention to what I am saying
67	Supervisor support	JCQ	My supervisor (or colleague) is helpful in getting the job done
68	Supervisor support	JCQ	My supervisor (or colleague) is successful in getting people to work together
69	Boredom	BPS	I would like more interesting things to do
70	Boredom	BPS	Sometimes, it happens I feel boring
71	Boredom	BPS	It takes a lot of change or variety to keep me really interested
72	Boredom	BPS	I find it easy to occupy myself
73	Boredom	BPS	I can usually find something to do or see to keep me interested
74	Monotony	FTBS	Many things I have to do are repetitive and monotonous
75	Monotony	FTBS	I have the feeling to always do the same thing
76	Monotony	FTBS	There is too much repetition in my activities
77	Lack of attention	MAAS	I find it difficult to stay focused on what's happening in the present
78	Lack of attention	MAAS	I do jobs or tasks automatically, without being aware of what I'm doing
79	Lack of attention	MAAS	It seems I'm “running on automatic” without much awareness of what I'm doing
80	Lack of attention	MAAS	I find myself doing things without paying attention
81	Lack of attention	MAAS	I do my activities quickly without attention
82	Environmental mastery	EMS	I am quite good at managing the responsibilities I have
83	Environmental mastery	EMS	I am good at juggling my time so that I can fit everything in that needs to get done
84	Environmental mastery	EMS	I often feel overwhelmed by my responsibilities
85	Environmental mastery	EMS	In general, I feel I have the control of the situation in which I am
86	Personal growth	PGS	I think it is important to have new experiences that challenge how you think about yourself and the world
87	Personal growth	PGS	I have the sense that I have developed as a person the last times
88	Personal growth	PGS	For me, this experience has been a continuous process of learning, changing, and growth
89	Personal growth	PGS	I like to realise that things have changed in good way that last months
90	Personal growth	PGS	The last times, I feel that I continue to learn more about myself as time goes by

**Fig. 1:**
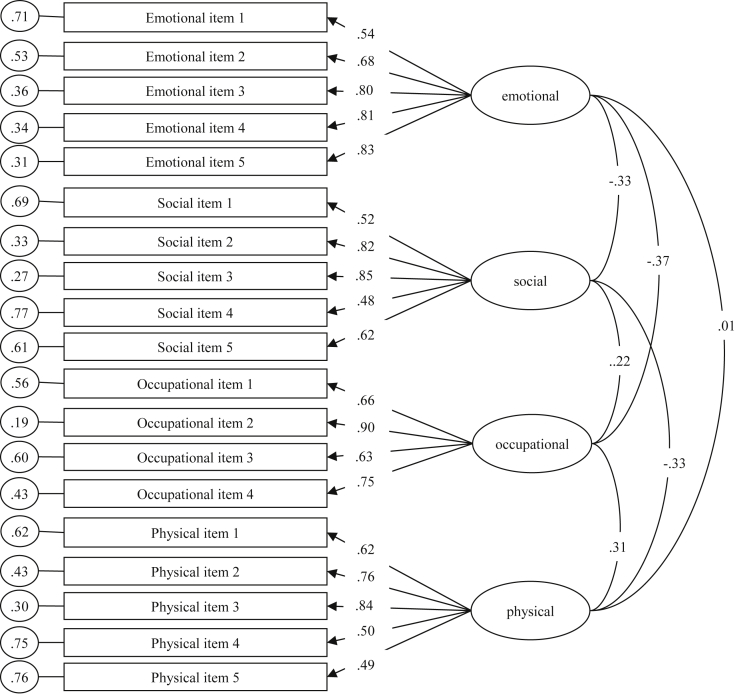
Results of confirmatory factor analysis of the ICE-Q scores (4-factor 19-item final version).

**Fig. 2:**
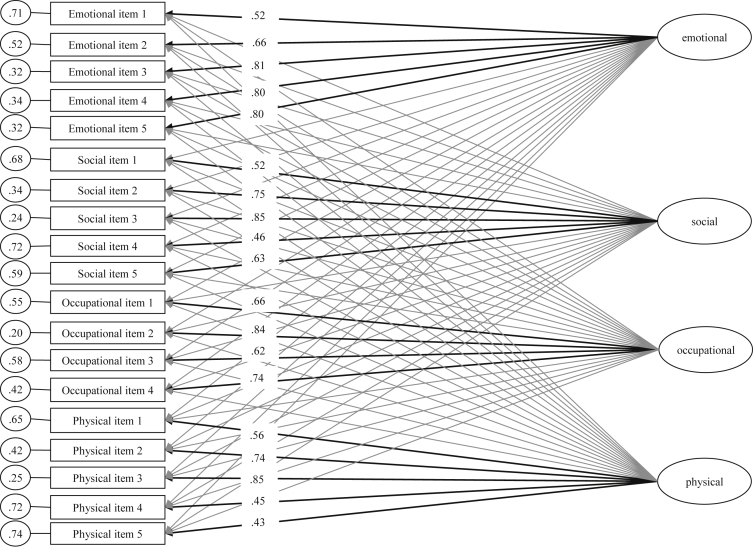
Results of exploratory structural equation modelling of the ICE-Q scores (4-factor 19-item final version). Notes. Black lines refer to the hypothesized paths between latent constructs and items whereas grey lines refer to the non-targeted paths between latent constructs and items. For ease of presentation, the hypothesized standardized factor loadings were presented whereas the non-targeted standardized factor loadings were not presented (*M*_non-targeted standardised factor loadings_ = 0.11, *SD* = 0.06, *MIN* = 0.01, *MAX* = 0.26).

Results of exploratory factor analysis with varimax rotation including all the items of the preliminary version of the ICE-Q are presented in Table 3. A four-factor solution was computed based on the scree test (the first four eigenvalues were substantially higher than the fifth one). Results of confirmatory factor analysis and exploratory structural equation modelling of the final 4-factor 19-item solution of the ICE-Q are presented in Fig. 1, Fig. 2 respectively. The four factors emerging from exploratory and confirmatory factor analyses were social (α = 0.82, *r*_test-retest over a 5-month period_ = .65), emotional (α = 0.85, *r* = 0.60), occupational (α = 0.82, *r* = 0.78) and physical (α = 0.78, *r* = 0.49).

**Table 3:** Results of exploratory factor analysis with varimax rotation including all the items of the preliminary version of the ICE-Q.

		Factor 1	Factor 2	Factor 3	Factor 4	*h*^*2*^
Item 1		–.15	.19	–.20	–.02	.10
Item 2		.13	.55	–.16	–.13	.36
Item 3		.00	.57	–.24	–.16	.41
Item 4		–.10	.48	–.24	.05	.30
Item 5		–.07	.63	–.30	–.01	.50
Item 6		–.17	.43	–.10	.11	.23
Item 7		–.16	.52	–.13	–.14	.33
Item 8		–.14	.58	.09	.07	.37
Item 9		–.19	.41	–.05	–.07	.21
Item 10		–.30	.62	.16	.18	.53
Item 11		–.31	.61	.16	.14	.51
Item 12		–.32	.59	.19	.19	.52
Item 13		–.14	.56	.11	.07	.35
Item 14		–.28	.44	.06	.02	.28
Item 15		–.30	.59	.13	–.01	.46
Item 16		–.16	.60	.13	.11	.42
Item 17		.03	.39	.23	.04	.21
Item 18		.19	.34	.23	.16	.23
Item 19		.08	.48	–.12	–.02	.25
Item 20		.04	.29	–.09	.05	.10
Item 21		.10	.49	–.20	.09	.30
Item 22		.19	–.04	.36	–.11	.18
Item 23		.09	–.13	.54	–.03	.32
Item 24		.17	–.13	.52	–.18	.36
Item 25		.35	–.11	.26	.13	.22
Item 26		.30	–.10	.25	.14	.19
Item 27		.43	.02	.08	.25	.26
Item 28		.35	–.39	.27	.14	.36
Item 29		.34	–.34	.17	.12	.27
Item 30		.23	–.42	.40	.03	.39
Item 31		.55	–.35	.18	.06	.47
Item 32		.58	–.40	.14	.10	.52
Item 33		.49	–.37	.17	.12	.42
Item 34		.63	–.16	.05	.13	.44
Item 35		.61	–.22	–.02	.09	.43
Item 36		.58	–.28	.13	.19	.46
Item 37		.71	.09	.11	–.27	.59
Item 38		.77	–.08	–.06	–.13	.62
Item 39		.73	.15	.06	.00	.56
Item 40		.78	.04	.04	–.10	.62
Item 41		.74	–.09	–.01	–.14	.57
Item 42		.59	.03	.03	–.08	.36
Item 43		.42	.08	.10	.05	.19
Item 44		.71	–.04	–.03	–.07	.50
Item 45		.68	.02	.06	–.08	.48
Item 46		.46	.29	.16	.24	.37
Item 47		–.40	.10	.07	.32	.28
Item 48		–.39	.30	.14	.46	.48
Item 49		–.55	.18	.09	.42	.52
Item 50		–.56	.26	.10	.38	.54
Item 51		.26	.27	.46	–.46	.56
Item 52		.18	.04	.59	.17	.42
Item 53		.15	.11	.49	.05	.27
Item 54		.15	.18	.46	–.54	.56
Item 55		.09	.12	.31	–.57	.45
Item 56		.18	.21	.45	–.43	.46
Item 57		.18	.40	.52	–.50	.71
Item 58		–.20	.48	.07	–.23	.33
Item 59		–.19	.41	.27	–.47	.49
Item 60		.03	.40	.40	–.50	.56
Item 61		.64	.03	.10	.13	.44
Item 62		.68	–.05	.10	.03	.47
Item 63		.62	–.05	.09	.02	.40
Item 64		.68	.00	.16	–.07	.49
Item 65		.57	–.13	.28	.01	.42
Item 66		.45	–.18	.29	.08	.32
Item 67		.52	–.08	.24	–.02	.34
Item 68		.57	–.12	.14	–.07	.36
Item 69		.05	.22	.16	.61	.45
Item 70		.23	.44	–.03	.52	.51
Item 71		.21	.16	.27	.45	.34
Item 72		.03	.00	–.61	.12	.39
Item 73		–.06	.05	–.58	.12	.36
Item 74		–.03	.14	.17	.77	.64
Item 75		.02	.34	.10	.72	.65
Item 76		–.04	.20	.05	.78	.65
Item 77		–.04	.50	–.25	.15	.34
Item 78		.02	.48	–.09	.24	.29
Item 79		.12	.46	–.05	.31	.33
Item 80		.15	.39	–.17	.44	.40
Item 81		.04	.46	–.14	.19	.27
Item 82		.04	–.18	.67	.01	.48
Item 83		.03	.07	.59	–.10	.37
Item 84		–.07	–.55	–.03	.00	.31
Item 85		.12	–.09	.67	–.00	.47
Item 86		.21	–.03	.47	.36	.39
Item 87		–.06	–.04	.67	.22	.50
Item 88		.06	–.19	.53	.00	.32
Item 89		.11	–.15	.65	.13	.47
Item 90		–.01	–.10	.63	.19	.44

Note. h^2^ = communalities.

Table 4, Table 5 displays the means and standard deviations of all the study variables for the 14 polar missions in sub-Antarctic stations (Amsterdam, Crozet and Kerguelen) and Antarctic stations (Concordia and Terre Adélie) respectively. Based on the rationale that the present paper focused on the presentation and description of the data, all the psychological variables were averaged (representing the mean score of the several measurement points) in order to obtain a unique score of each psychological construct for each of the 14 polar missions. Finally, the final version of the ICE-Q is included in Table 6.

**Table 4:** Descriptive statistics for all the study variables across each of the 9 polar missions in Sub-Antarctic stations (Amsterdam, Crozet, Kerguelen).

Polar missions	Amsterdam 11–12 (n = 14)		Amsterdam 12–13 (n = 19)		Amsterdam 13–14 (n = 40)		Crozet 11–12 (n = 21)		Crozet 12–13 (n = 13)		Crozet 13–14 (n = 38)		Kerguelen 11–12 (n = 15)		Kerguelen 12–13 (n = 7)		Kerguelen 13–14 (n = 43)	
	*M*	*SD*	*M*	*SD*	*M*	*SD*	*M*	*SD*	*M*	*SD*	*M*	*SD*	*M*	*SD*	*M*	*SD*	*M*	*SD*
Physical (ICE-Q)	2.54	0.65	2.40	0.38	2.35	0.68	2.58	0.52	2.89	0.77	2.58	0.67	2.12	0.35	2.29	0.43	2.29	0.56
Social3 (ICE-Q)	3.51	0.52	3.29	0.81	3.89	0.76	4.37	0.56	3.23	0.85	3.37	0.56	3.83	0.62	5.17	0.62	4.27	0.60
Occupational (ICE-Q)	2.68	0.88	2.69	0.94	2.33	0.99	3.31	1.29	3.10	1.06	2.43	0.86	2.60	0.72	1.07	0.12	3.10	0.73
Psychological (ICE-Q)	2.43	0.62	2.07	0.88	2.84	0.73	2.42	0.91	2.35	0.87	2.59	0.53	2.38	0.94	2.37	0.57	2.36	0.80
Stress (RESTQ)	2.14	0.50	1.66	0.38	2.13	0.56	2.09	0.55	2.43	0.68	2.19	0.37	1.83	0.50	2.13	0.30	2.06	0.41
Recovery (RESTQ)	4.11	0.54	4.64	0.35	4.51	0.46	4.42	0.49	4.16	0.56	4.14	0.39	4.30	0.49	4.88	0.17	4.59	0.42
Cohesiveness (GES)	3.80	0.63	3.23	0.77	4.03	0.80	4.50	0.57	3.34	0.88	3.54	0.62	3.96	0.82	5.26	0.65	4.45	0.61
Implementation/preparedness (GES)	3.47	0.44	3.43	0.78	4.02	0.64	4.22	0.80	3.18	0.76	3.33	0.49	3.76	0.51	4.29	0.30	4.16	0.59
Counterproductive activity GES	1.93	0.83	2.05	1.16	1.84	0.78	2.07	0.85	1.98	0.92	1.79	0.74	2.43	0.68	1.25	0.32	1.51	0.49
Decision latitude (JCQ)	3.79	1.04	4.32	0.83	3.96	0.73	4.67	1.07	4.40	1.10	3.76	0.59	4.17	1.09	4.40	0.16	4.36	0.73
Psychological job demands (JCQ)	2.53	0.76	2.54	0.90	2.18	0.86	3.02	1.15	2.94	0.95	2.26	0.75	2.57	0.59	1.11	0.11	2.79	0.61
Social support from colleagues (JCQ)	4.52	0.36	4.71	0.83	4.71	0.64	5.29	0.58	4.87	0.83	4.38	0.88	4.40	0.92	5.79	0.17	4.96	0.61
Social support from supervisor (JCQ)	4.20	1.39	4.37	0.81	4.06	0.97	5.18	0.76	4.23	1.04	3.77	1.33	3.70	1.24	5.57	0.31	3.92	1.17
Depressive symptoms (BDI-II)	2.71	2.40	1.91	1.81	–	–	5.10	4.25	4.09	2.43	–	–	3.13	2.07	1.00	1.73	–	–
Boredom (BPS)	2.43	0.65	2.19	0.78	2.56	0.81	2.11	0.62	2.32	0.51	2.50	0.64	2.35	0.70	2.57	0.35	2.53	0.65
Monotony (FTBS)	2.52	0.68	2.14	0.90	2.90	0.84	2.48	1.00	2.33	0.92	2.67	0.68	2.27	1.10	2.10	0.60	2.38	0.83
Lack of attention (MAAS)	2.31	0.67	1.52	0.45	1.85	0.77	1.88	0.69	1.55	0.46	1.86	0.60	2.04	0.91	1.40	0.31	1.93	0.71
Environment mastery (PWBS)	4.52	0.62	4.75	0.53	4.85	0.63	5.02	0.67	4.31	0.74	4.45	0.93	4.98	0.61	5.46	0.30	4.74	0.55
Personal growth (PWBS)	3.94	1.43	4.73	1.05	4.20	0.96	4.57	0.75	4.29	0.70	3.77	0.79	3.72	0.36	4.97	0.51	4.20	0.73
Primary appraisal	1.98	0.77	1.50	0.59	1.95	0.79	2.11	0.82	2.42	0.92	2.08	0.81	2.16	0.70	1.64	0.52	1.91	0.75
Secondary appraisal	4.50	0.56	4.89	0.51	4.70	0.71	5.14	0.50	4.49	0.98	4.61	0.69	4.73	0.78	5.43	0.31	4.81	0.57
Optimism (LOT-R)	4.50	0.67	4.50	0.82	4.33	0.76	4.71	0.58	4.36	0.74	3.99	0.58	4.77	0.64	5.57	0.44	4.71	0.63
Coping strategies (brief COPE)																		
Self-distraction	3.14	0.72	2.45	0.83	3.16	1.14	3.13	1.11	2.92	0.86	2.79	1.10	3.43	0.65	2.86	0.38	2.83	1.26
Active coping	2.57	1.04	4.53	1.30	4.11	0.93	2.60	0.82	4.19	1.05	3.80	1.23	2.46	0.95	4.14	0.94	3.76	0.85
Denial	2.89	0.81	1.18	0.42	1.81	0.83	3.12	1.19	1.77	0.78	1.42	0.56	3.57	0.83	1.93	0.73	1.93	0.87
Substance use	2.50	1.06	1.24	0.42	1.54	0.89	2.90	0.98	1.92	0.81	1.92	0.88	3.21	0.70	1.00	0.00	1.44	0.59
Use of emotional support	2.32	1.25	3.18	1.36	2.60	0.71	2.10	0.78	2.92	0.93	3.07	1.03	2.04	0.60	2.64	0.48	2.48	1.04
Use of instrumental support	3.89	0.84	3.61	1.16	2.90	0.96	4.10	0.93	3.88	0.89	3.34	1.16	4.21	0.61	3.57	1.54	3.01	0.79
Behavioral disengagement	2.79	0.61	1.16	0.37	1.46	0.70	2.81	0.98	1.88	0.98	1.37	0.45	2.93	0.76	1.14	0.38	1.45	0.54
Venting	2.54	0.63	2.76	1.35	2.56	0.65	2.19	0.81	3.08	0.81	3.20	1.01	2.61	0.86	2.36	0.56	2.52	0.80
Positive reframing	3.43	0.85	3.79	1.65	3.46	1.29	3.24	1.16	4.35	1.01	3.57	1.18	3.46	0.66	4.00	1.53	3.83	1.40
Planning	2.57	0.92	4.21	0.90	3.79	0.98	2.29	0.85	3.88	1.19	3.54	1.18	2.71	0.70	4.93	0.89	3.93	0.77
Humor	3.36	0.86	3.97	0.92	3.46	1.24	3.29	1.21	3.65	1.48	3.66	1.06	3.54	0.69	4.57	0.79	4.18	0.87
Acceptance	3.36	0.86	4.21	1.17	4.40	0.99	4.02	1.28	4.69	0.95	3.86	1.09	3.79	1.01	5.71	0.49	4.53	0.69
Religion	1.50	0.39	1.53	0.95	1.49	0.91	1.40	0.66	1.00	0.00	1.36	0.73	1.29	0.43	1.07	0.19	1.65	1.14
Self-blame	3.54	0.95	2.45	0.86	2.97	0.89	4.00	1.55	2.77	1.13	2.47	0.72	3.93	0.96	1.07	0.19	2.76	0.77
Defense mechanisms (DSQ)																		
Displacement	1.82	0.70	1.16	0.47	1.70	0.74	2.07	1.09	1.81	0.52	1.64	0.70	1.68	0.67	1.21	0.27	1.89	0.77
Acting out	2.36	1.12	1.45	0.52	1.98	0.86	3.26	1.52	2.62	1.00	2.22	0.95	2.32	0.77	1.07	0.19	2.04	0.86
Passive aggressiveness	1.68	0.50	1.11	0.32	1.49	0.79	1.71	0.75	1.77	0.67	1.36	0.48	1.71	0.61	1.07	0.19	1.35	0.43
Undoing	1.50	0.55	1.08	0.19	1.58	0.69	1.69	0.68	2.08	0.95	1.74	0.72	1.62	0.89	1.07	0.19	1.35	0.51
Projection	1.79	0.70	1.18	0.38	1.55	0.72	1.60	0.70	1.62	0.22	1.46	0.47	1.57	0.68	1.00	0.00	1.29	0.41
Splitting of other	2.39	0.88	1.11	0.21	2.33	0.92	2.05	0.93	2.00	0.68	1.64	0.79	2.00	1.18	1.00	0.00	1.92	0.72
Rationalization	3.07	0.83	3.16	0.47	3.03	0.64	3.38	0.72	3.08	0.79	2.64	0.53	3.46	0.66	2.79	0.49	3.19	0.58
Denial	2.68	1.17	1.55	0.55	2.23	0.71	2.17	0.59	2.42	0.70	2.12	0.72	2.50	1.06	1.64	0.94	2.25	0.99
Dissociation	2.29	0.85	1.55	0.50	2.33	1.00	1.98	0.80	1.96	0.95	1.91	0.82	2.18	0.77	1.00	0.00	2.31	0.96
Devaluation of other	1.32	0.42	1.03	0.11	1.51	0.66	1.31	0.43	1.31	0.60	1.47	0.58	1.46	0.37	1.00	0.00	1.33	0.45
Fantasy	2.04	0.80	1.18	0.38	1.83	0.84	1.79	0.99	1.92	0.86	1.67	0.65	1.68	0.80	1.00	0.00	1.66	0.58
Isolation	2.75	0.91	1.84	0.58	2.97	1.07	2.88	1.09	2.23	0.56	2.20	0.77	3.11	0.81	1.00	0.00	2.90	0.70
Altruism	4.04	1.23	4.63	0.57	4.14	0.99	4.43	1.09	4.77	1.01	3.54	0.84	4.43	0.73	5.21	0.57	3.86	1.02
Reaction formation	2.71	0.89	3.42	1.29	3.16	1.08	3.90	1.16	3.54	1.14	3.27	0.85	2.89	1.13	3.86	1.11	3.26	0.89
Suppression	2.93	0.68	2.11	0.76	2.76	0.94	3.52	0.73	3.92	0.73	2.30	0.79	2.86	0.89	2.71	1.52	2.68	0.79
Idealization	1.96	1.10	1.18	0.38	2.43	1.29	2.14	0.94	2.58	1.22	1.73	0.68	1.96	0.93	1.00	0.00	2.09	0.76
Repression	3.61	0.92	4.11	0.86	3.99	1.00	4.38	1.14	3.92	0.76	3.09	0.99	4.11	0.86	4.50	1.04	4.06	0.78
Humor	3.93	1.04	4.45	0.88	4.61	0.83	4.33	1.04	4.69	1.01	4.03	0.94	4.46	0.77	5.29	0.39	4.16	0.79
Anticipation	3.36	0.74	3.76	0.93	3.34	0.75	4.29	0.98	4.27	0.81	3.09	0.95	4.04	1.08	3.36	1.52	3.70	0.82
Sublimation	3.00	0.94	3.82	0.75	3.54	1.00	3.74	1.00	3.73	0.86	2.90	1.12	3.25	1.07	3.79	1.11	3.27	1.00

Notes. ICE-Q = isolated and confined environments questionnaire, GES = group environment scale, JCQ = job content questionnaire, RESTQ = Recovery Stress Questionnaire, BPS = boredom proneness scale, FTBS = free time boredom scale, MAAS = mindfulness attention awareness scale, PWBS = psychological well-being scales, COPE = multidimensional coping inventory, DSQ = defense style questionnaire, BDI-II = Beck depression inventory-II, LOT-R = life orientation test-revised.

**Table 5:** Descriptive statistics for all the study variables across each of the 5 polar missions in Antarctic stations (Concordia and Terre Adélie).

polar missions	Concordia 10–11 (n = 83)		Concordia 12–13 (n = 69)		Concordia 13–14 (n = 32)		Terre Adélie 12–13 (n = 48)		Terre Adélie 13–14 (n = 37)	
	*M*	*SD*	*M*	*SD*	*M*	*SD*	*M*	*SD*	*M*	*SD*
Physical (ICE-Q)	2,68	0,70	2,82	0,68	3,15	0,79	2,78	0,83	2,98	0,76
Social (ICE-Q)	3,63	0,95	3,15	0,82	3,04	0,94	3,95	0,64	3,84	0,66
Occupational (ICE-Q)	2,85	1,06	3,17	0,97	3,21	1,09	3,08	1,20	2,52	0,81
Psychological (ICE-Q)	2,92	1,03	2,43	0,82	2,45	0,73	2,77	0,91	2,60	0,68
Stress (RESTQ)	2,07	0,53	2,22	0,51	2,15	0,44	2,17	0,65	2,17	0,48
Recovery (RESTQ)	4,29	0,58	4,06	0,53	3,85	0,73	4,42	0,68	4,02	0,60
Cohesiveness (GES)	3,61	0,91	3,27	0,89	3,19	0,93	3,94	0,61	4,02	0,70
Implementation/preparedness (GES)	3,80	0,90	3,12	0,79	3,08	0,77	4,19	0,55	3,83	0,57
Counterproductive activity GES	2,24	1,06	2,44	1,04	2,13	1,03	1,43	0,41	1,38	0,44
Decision latitude (JCQ)	4,30	0,74	4,22	1,03	4,03	0,90	4,16	1,31	3,63	0,83
Psychological job demands (JCQ)	2,62	0,98	2,91	0,94	3,07	0,99	2,85	0,95	2,33	0,69
Social support from colleagues (JCQ)	4,44	0,82	4,10	1,04	3,89	1,08	4,88	0,73	4,24	0,96
Social support from supervisor (JCQ)	3,99	1,32	3,75	1,19	3,06	1,20	4,14	1,02	3,84	0,90
Depressive symptoms (BDI-II)	3,13	3,01	4,32	3,81	–	–	5,65	5,29	–	–
Boredom (BPS)	2,93	0,58	2,50	0,66	2,48	0,60	2,40	0,92	2,44	0,55
Monotony (FTBS)	2,81	1,13	2,37	0,98	2,39	0,82	2,86	0,95	2,76	0,88
Lack of attention (MAAS)	1,78	0,58	1,63	0,57	1,60	0,54	1,78	0,69	1,96	0,60
Environment mastery (PWBS)	4,80	0,58	4,87	0,69	4,60	0,64	4,76	0,83	4,44	0,56
Personal growth (PWBS)	4,05	0,95	4,29	1,02	3,70	0,73	4,04	1,16	3,71	0,72
Primary appraisal	1,83	0,84	1,99	0,75	2,01	0,83	2,15	0,86	1,94	0,57
Secondary appraisal	4,91	0,54	4,86	0,60	4,85	0,78	4,85	0,73	4,62	0,59
Optimism (LOT-R)	4,59	0,64	4,50	0,72	4,13	0,54	4,10	0,80	4,05	0,67
Coping strategies (brief COPE)										
Self-distraction	2,86	0,88	3,38	1,10	2,58	1,19	2,88	0,92	2,49	1,02
Active coping	2,85	0,98	4,28	1,04	3,52	1,15	3,85	1,42	3,76	1,35
Denial	2,98	0,78	1,70	0,80	1,38	0,68	1,93	0,85	1,53	0,60
Substance use	2,48	0,95	1,57	1,01	1,22	0,47	1,84	1,18	1,25	0,50
Use of emotional support	1,46	0,52	2,61	1,06	2,42	1,05	3,09	1,45	2,58	1,07
Use of instrumental support	3,49	0,89	2,86	1,23	2,86	0,94	3,11	1,17	2,92	1,16
Behavioral disengagement	2,95	0,88	1,52	0,78	1,20	0,44	1,76	0,72	1,50	0,77
Venting	1,74	0,66	2,44	1,05	2,45	1,15	2,93	1,40	2,50	1,02
Positive reframing	2,80	1,01	4,07	1,13	3,59	1,52	3,66	1,39	3,26	1,20
Planning	2,01	1,08	4,13	1,16	3,33	1,05	3,76	1,48	3,75	1,19
Humor	3,11	0,96	3,47	1,17	3,47	1,34	3,09	1,34	3,10	1,12
Acceptance	3,65	1,33	4,28	1,22	4,09	1,51	4,30	1,48	4,17	1,13
Religion	1,59	1,13	1,93	1,30	1,63	1,09	1,31	0,60	1,74	1,11
Self-blame	3,73	1,20	2,25	0,98	2,05	0,78	2,35	1,00	2,38	0,72
Defense mechanisms (DSQ)										
Displacement	1,72	0,78	1,72	0,82	1,59	0,70	2,03	1,21	1,65	0,50
Acting out	2,51	0,81	2,24	0,88	1,97	0,73	2,63	1,36	2,12	0,75
Passive aggressiveness	1,55	0,70	1,62	0,71	1,69	0,66	1,36	0,43	1,65	0,48
Undoing	1,30	0,55	1,49	0,65	1,38	0,58	1,61	0,81	1,72	0,57
Projection	1,40	0,49	1,62	0,62	1,52	0,55	1,45	0,61	1,46	0,36
Splitting of other	1,88	0,80	2,09	0,92	1,59	0,72	1,96	0,95	1,59	0,58
Rationalization	3,28	0,86	3,16	0,80	3,58	1,12	3,11	0,82	2,88	0,58
Denial	1,82	0,67	2,26	0,92	2,11	0,81	1,97	0,64	2,07	0,72
Dissociation	2,37	1,31	2,34	1,04	2,08	1,15	2,30	0,85	1,95	0,86
Devaluation of other	1,26	0,55	1,41	0,60	1,25	0,54	1,51	0,66	1,36	0,42
Fantasy	1,57	0,92	1,61	0,74	1,47	0,67	1,60	0,70	1,68	0,72
Isolation	2,51	1,30	2,24	0,96	2,42	0,85	2,46	0,99	2,55	1,01
Altruism	4,10	1,02	3,54	1,09	3,33	0,95	3,63	0,95	3,78	0,87
Reaction formation	2,66	0,96	3,01	1,19	2,81	0,81	2,79	0,86	3,19	0,77
Suppression	2,25	1,02	2,64	1,08	2,16	0,84	2,70	1,00	2,46	0,84
Idealization	1,71	0,82	2,19	1,23	1,42	0,62	2,42	0,97	1,88	0,90
Repression	3,46	1,03	3,68	0,83	3,59	0,90	3,62	0,80	3,62	0,72
Humor	4,35	0,95	4,15	1,07	4,41	1,04	3,93	1,26	3,66	0,55
Anticipation	3,56	1,15	3,63	0,90	3,50	0,88	3,50	1,05	3,72	0,74
Sublimation	3,45	1,18	3,70	1,17	2,84	1,01	3,49	1,28	3,12	0,99

Notes. ICE-Q = isolated and confined environments questionnaire, GES = group environment scale, JCQ = job content questionnaire, RESTQ = Recovery Stress Questionnaire, BPS = boredom proneness scale, FTBS = free time boredom scale, MAAS = mindfulness attention awareness scale, PWBS = psychological well-being scales, COPE = multidimensional coping inventory, DSQ = defense style questionnaire, BDI-II = Beck depression inventory-II, LOT-R = life orientation test-revised.

**Table 6:** ICE-Q questionnaire.

	1 never	2 seldom	3 sometimes	4 often	5 more often	6 always
1. Group members can understand what others in the group are going through	1	2	3	4	5	6
2. My job requires working very fast	1	2	3	4	5	6
3. I did not get enough sleep	1	2	3	4	5	6
4. I would like more interesting things to do	1	2	3	4	5	6
5. Group members are supportive of one another	1	2	3	4	5	6
6. My job requires working very hard	1	2	3	4	5	6
7. I feel physically relaxed	1	2	3	4	5	6
8. Many things I have to do are repetitive and monotonous	1	2	3	4	5	6
9. Group activities are easy to follow	1	2	3	4	5	6
10. I am asked to do too much work	1	2	3	4	5	6
11. I am dead tired after work	1	2	3	4	5	6
12. Sometimes, I feel bored	1	2	3	4	5	6
13. Group members learn new ways of solving problems	1	2	3	4	5	6
14. My job requires long periods of intense concentration on the task	1	2	3	4	5	6
15. I feel physically fit	1	2	3	4	5	6
16. I feel that I am doing the same thing over and over	1	2	3	4	5	6
17. Group members encourage each other in reaching their goals	1	2	3	4	5	6
18. I am overtired	1	2	3	4	5	6
19. There is too much repetition in my activities	1	2	3	4	5	6

## 2 Experimental design, materials, and methods

Participants were winterers from several sub-Antarctic and Antarctic stations. The study protocol was carried out in accordance with the Declaration of Helsinki and was approved by the European space agency, the Paul Emile Victor institute and the local institutional review board. After comprehensive verbal and written explanations of the study, all the subjects gave their written informed consent to participate. Participants were asked to return the completed questionnaires directly to the researchers within two days after receiving the battery in their personal email account. The questionnaires were sent and returned the first days that the respondents were on site and at 3, 5, 6, 8, 10, and/or 12 months into the mission.

A battery of well-known and validated questionnaires was used to record the winterers' responses to polar stations. Because of the high workloads and psychological challenges of isolated, confined and extreme environments, shortened versions of the original scales were sometimes used. Participants completed the group environment scale (GES) [2] measuring cohesiveness, implementation and preparedness, and counterproductive activity, the job content questionnaire (JCQ) [3] assessing decision latitude, psychological job demands, social support from colleagues and supervisor, the Recovery Stress Questionnaire (RESTQ) [4] measuring balance between stress and recovery from physical, emotional, behavioral, and social perspectives, the boredom proneness scale (BPS) [5] assessing constraint, affective responses, perception of time, external and internal stimulation, the free time boredom scale (FTBS) [6] measuring the individual's perception of boredom in periods of leisure, the mindfulness attention awareness scale (MAAS) [7] assessing the cognitive, emotional, physical, and interpersonal domains of awareness in the present moment, the environment mastery scale and personal growth scale, retrieved from psychological well-being scales (PWBS) [8] assessing the capacity to effectively manage one's life and the surrounding environment and the individuals' perception of continuing personal development and openness to new experiences, a short questionnaire measuring primary and secondary appraisals [9], The brief COPE [10] assessing a wide variety of coping strategies used to deal with stress (acceptance, active coping, behavioral disengagement, denial, humor, planning, positive reframing, religious, self-blame, self-distraction, substance use, using emotional support, using instrumental support and venting), the defense style questionnaire [11] assessing a wide variety of individual's conscious derivatives of defense mechanisms (acting-out, altruism, anticipation, denial, devaluation of other, displacement, dissociation, fantasy, humor, idealization, isolation, passive aggressive, projection, rationalization, reaction formation, repression, splitting of other, sublimation, suppression, undoing), the Beck depression inventory-II [12] measuring depressive symptoms such as sadness, crying, and indecisiveness, and the life orientation test-revised [13] assessing dispositional optimism. It is noteworthy that the items of the preliminary version of the ICE-Q were comprised of the GES, JCQ, RESTQ, BPS, FTBS, MAAS, and PWBS items.
